# Electrocorticographic Temporal Alteration Mapping: A Clinical Technique for Mapping the Motor Cortex with Movement-Related Cortical Potentials

**DOI:** 10.3389/fnins.2017.00326

**Published:** 2017-06-12

**Authors:** Zehan Wu, Tao Xie, Lin Yao, Dingguo Zhang, Xinjun Sheng, Dario Farina, Liang Chen, Ying Mao, Xiangyang Zhu

**Affiliations:** ^1^Department of Neurosurgery, Huashan Hospital, Fudan UniversityShanghai, China; ^2^State Key Laboratory of Mechanical System and Vibration, Shanghai Jiao Tong UniversityShanghai, China; ^3^Department of Systems Design Engineering, Faculty of Engineering, University of WaterlooWaterloo, ON, Canada; ^4^Department of Bioengineering, Imperial College LondonLondon, United Kingdom

**Keywords:** intraoperative, electrocorticography (ECoG), motor cortex mapping, movement-related cortical potentials (MRCP)

## Abstract

We propose electrocorticographic temporal alteration mapping (ETAM) for motor cortex mapping by utilizing movement-related cortical potentials (MRCPs) within the low-frequency band [0.05-3] Hz. This MRCP waveform-based temporal domain approach was compared with the state-of-the-art electrocorticographic frequency alteration mapping (EFAM), which is based on frequency spectrum dynamics. Five patients (two epilepsy cases and three tumor cases) were enrolled in the study. Each patient underwent intraoperative direct electrocortical stimulation (DECS) procedure for motor cortex localization. Moreover, the patients were required to perform simple brisk wrist extension task during awake craniotomy surgery. Cross-validation results showed that the proposed ETAM method had high sensitivity (81.8%) and specificity (94.3%) in identifying sites which exhibited positive DECS motor responses. Moreover, although the sensitivity of the ETAM and EFAM approaches was not significantly different, ETAM had greater specificity compared with EFAM (94.3 vs. 86.1%). These results indicate that for the intraoperative functional brain mapping, ETAM is a promising novel approach for motor cortex localization with the potential to reduce the need for cortical electrical stimulation.

## 1. Introduction

With the development of anesthesia technique, awake craniotomy surgery has been widely used for eloquent cortex mapping (Bennett et al., 1997). During the surgery, surgeons are able to locate the brain functional area using direct electrocortical stimulation (DECS) in order to accurately resect the lesion while minimizing post-surgical functional impairments. DECS is widely considered as the “golden standard” for intraoperatively delineating eloquent cortex, but it is a misconception that DECS allows us to draw unequivocal conclusions about the role of stimulated brain areas (Borchers et al., 2011). The DECS meets its burden because of time-consuming and high risk of inducing seizures (Nader et al., 2004). To further provide a high-quality brain surgery and overcome those unavoidable disadvantages, electrocorticographic (ECoG) signal changes associated with the function of specific cortical area has received substantial interest as an alternative approach for the eloquent cortex localization (Ikeda et al., 2002).

The recorded ECoG electrical potential at a broad range of frequencies is illustrated to be strongly correlated with brain activity. The frequency component normally comprises of slow cortical potential (e.g., movement-related cortical potentials [0.05–3] Hz), low frequency oscillation (mu [8–12] Hz, beta [18–26] Hz), and high frequency oscillation (>30 Hz). Each of these components has the potential for cortical function mapping as they can be used to quantify brain activity from a different point-of-view (Ikeda et al., 2002; Leuthardt et al., 2007; Miller et al., 2007b). Low frequency oscillations are produced by thalamo-cortical circuits and their amplitude decreases when brain is activated, such as when motor tasks are performed (Pfurtscheller and Silva, 1999). In contrast, high frequency changes are found to have an increase in amplitude with motor tasks and are more correlated with the activity of local neuronal populations (Manning et al., 2009). Specifically, high-frequency tends to have more concentrated spatial origin than low-frequency (Leuthardt et al., 2007). Within high frequency bands, several studies have shown that it was highly specific to cortical processing in motor tasks (Miller et al., 2007b). Clinically, Leuthardt et al. first used the term of electrocorticographic frequency alteration mapping (EFAM), including both low frequency band (LFB) and high frequency band (HFB) alteration, for the purpose of motor cortex regions mapping (Leuthardt et al., 2007). It has shown that HFB sites were more task specific, whereas LFB sites more sensitive in identifying functional cortex defined by DECS (Miller et al., 2007a; Breshears et al., 2010; Roland et al., 2010), and a combination of HFB and LFB is necessary for specific function localization, such as Broca localization (Wu et al., 2010).

However, high frequency activation is task specific and different tasks generate different activity patterns, e.g., power increases in gamma band was difficult to detect when performing only simple wrist movement (Aoki et al., 1999). Generally, in intraoperative condition, it is hard and time-consuming for patients to perform complex motor task, while a simple movement task, such as wrist extension and flexion, would be an ideal choice for corresponding motor cortex mapping. As no significant gamma activity was induced with simple wrist movement in our study, movement-related cortical potentials (MRCPs) were used for motor function localization.

Cortical potentials associated with voluntary movements are identified as MRCPs since the pioneering work of Bates (1951) and Vaughan et al. (1968). Components of MRCPs are related to the early preparation (Bereithschaftspotential or readiness potential), late preparation (negative slope, NS), initiation (motor potential, MP), and execution of the movement (movement-related response 1, MRR1) (Babiloni et al., 1999). Careful analysis of MRCPs enables us to evaluate the efferent function of the brain and higher brain functions controlling voluntary movements. MRCPs mainly depend on cerebellar-thalamus-cortical circuitry (Shibasaki et al., 1986; Tarkka et al., 1993), and can be used for primary and supplementary motor area function mapping (Ikeda et al., 2002). For different types of movements (e.g., hand, finger, foot, tongue protrusions, and vocalizations), MRCPs have a specific topographic distribution on the motor cortex, moreover, the distribution is consistent with the electrical simulation results in humans (Fried et al., 1981; Neshige et al., 1988; Ikeda et al., 1992, 1995a,b; Yazawa et al., 1997). These studies indicate MRCPs would be a clinically viable method for precise motor cortical mapping.

The MRCP is regarded as the amplitude changing of the [0.05–3] Hz slow cortical potential within the temporal domain. In this study, we defined a term, i.e., electrocorticographic temporal alteration mapping (ETAM), when applying the temporal information for eloquent cortex mapping. Although, MRCPs were selected as the ETAM signal feature in this paper, other researchers could apply specific event-related potentials as the ETAM signal feature for sensory, cognitive, or language cortex mapping.

ECoG-based mapping predominantly occurs in the epilepsy monitoring unit, remaining as an extraoperative endeavor. However, only a few attempts tried to use ECoG in the intraoperative settings instead of after-surgery monitoring for motor and speech/language cortex localization (Breshears et al., 2010; Roland et al., 2010; Ogawa et al., 2014; Taplin et al., 2016). The practicality and potential value of ECoG-based mapping in the operating room is still largely unexplored. In this study, the proposed ETAM method was tested and validated on patients undergoing intraoperative brain surgery, and compared with the results from clinically-proofed DECS method and the state-of-the-art EFAM method.

## 2. Materials and methods

### 2.1. Subjects

Five patients (two males and three females, two epilepsy and three brain tumors, all right-handed) underwent standard awake craniotomies with DECS cortical mapping and focus resection. The corresponding information of each patients was presented in Table 1. After dural opening and hemostasis, ECoG electrode arrays were placed on the surface of the brain to record surface cortical potentials. Arrays were covered by wet laps to maintain contact between the electrodes and the cortical surface. The wet laps also prevented array shift during the mapping process. All subjects recruited by Huashan Hospital were informed about the whole experiment. The study was approved by the Ethics Committee of Huashan Hospital. All subjects have signed the informed consent forms by themselves and immediate family.

**Table 1:** Clinical summary of patients enrolled in the study.

**Sub**.	**Age**	**Gender**	**Moving hand**	**Cognitive**	**Tumor/epilepsy Focus**
1	26	F	R	Normal	L temporal epilepsy
2	40	F	R	Normal	L temporal tumor
3	32	F	L	Normal	R temporal epilepsy
4	51	M	R	Normal	L temporal tumor
5	53	M	R	Normal	L temporal tumor

*Normal: non-motion disorder*.

### 2.2. Recordings

Platinum electrode arrays (PMT, USA) were typically configured as linear strips or 4 × 8, 6 × 8 electrode arrays. All electrodes had 4 mm in diameter, 1 cm inter-electrode distance, and were embedded in SILASTIC. All signals were recorded using Synamps2 system (Neuroscan, USA) with an analog bandwidth filter of DC to 200 Hz and a notch filter of 50 Hz. Signals were digitally sampled at 2,000 Hz. A pair of surface electromyography (EMG) electrodes were mounted on the wrist extensor muscle of the contralateral hand. Surface EMG signals were recorded in bipolar derivation and sampled simultaneously with the ECoG signal using the same amplifier. Needle electrodes at the contralateral and ipsilateral mastoid were set as reference and ground, respectively.

### 2.3. Tasks

When patients were considered to be awake and conscious, they were informed to execute single brisk wrist extension motion according to the voice cue provided by the surgeons. During a trial, patients were instructed to execute the motion as fast as possible and relaxed with normal speed after holding contraction for about 1 s. Forty trials were performed by each patient. The trial intervals were randomly between 5 and 16 s. During the measurement, patients were instructed to close their eyes and focus attention on the hand moving.

### 2.4. Signal analysis

We excluded channels containing lots of artifacts because of faulty connections. Then the data from each remaining channel were referenced to common averaged reference (CAR). The onset of the electromyographic (EMG) response was used as the starting time, which was visually identified (Ikeda et al., 1992).

#### 2.4.1. Electrocorticographic temporal alteration mapping, ETAM

ECoG signals were band-pass filtered with a 2nd order Butterworth filter of [0.05–3] Hz. Each trial was extracted 2,000 ms before and 4,000 ms after the EMG onset (zero time). The grand-average MRCP waveform for each channel was obtained by averaging all the trials. The baseline for each grand-average MRCP waveform was determined as the mean of the first 400 ms, i.e., [2,000–1600] ms before the EMG onset. For each trial, we defined the interval from 0 to 500 ms [motor potential and movement-related response (Babiloni et al., 1999)] as task-component and the interval from –2,000 to -1,500 ms as rest-component. The ETAM method included the following three steps: (1) calculating the MRCP template; (2) for each trial, calculating the correlation coefficient between the template and the task-component (or rest-component); (3) for each channel, calculating the *R*^2^ index between task and rest.

**Figure 1:**
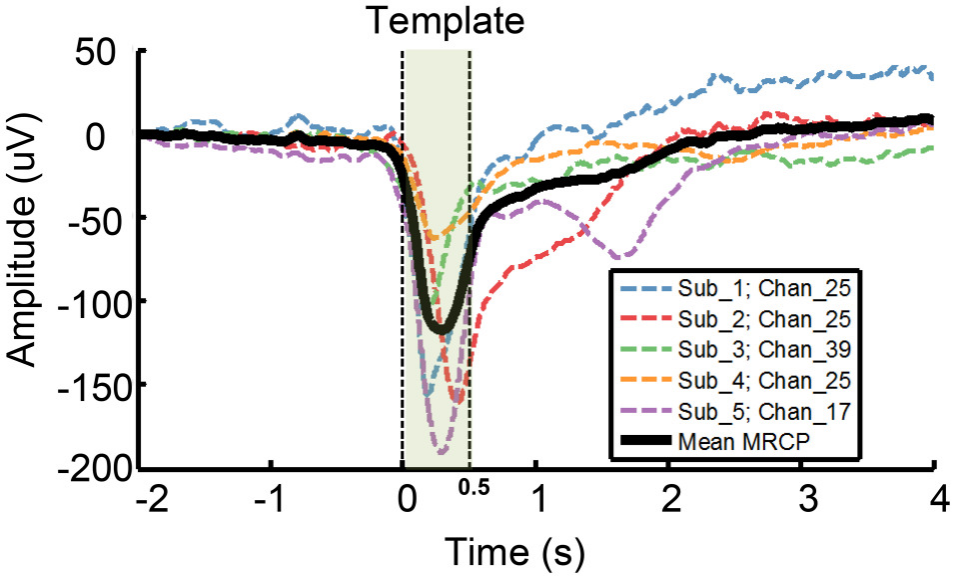
Template used for ETAM method. A representative grand average MRCP waveform was chosen from each subject, i.e., channel 25,25,39,25,17 from subject 1–5, respectively. Each dash line indicates a single channel grand average MRCP waveform, and the bold solid line, i.e., the Mean MRCP, indicates the average of the five representative channel MRCPs. The template (shadow area) is a 500 ms segment of the mean MRCP from 0 to 500 ms.

- Step 1: We chose a representative grand average MRCP waveform with largest amplitude from each subject (channel 25, 25, 39, 25, 17 from subject 1–5, respectively. Dash line in Figure 1), and calculated the mean MRCP (bold solid line in Figure 1). The template was a 500 ms segment of the mean MRCP (from 0 to 500 ms, including MP and MRR1, shadow area in Figure 1).
- Step 2: For each trial, we calculated the correlation coefficient (*r*) between the template and the task-component resulting the task-CC (task correlation coefficient), and the correlation coefficient between the template and the rest-component resulting the rest-CC (rest correlation coefficient), shown in Equation (1). A higher *r*-value indicates a better degree of waveform similarity between the ECoG component and the template.
  (1) $$\begin{array}{r} r=\frac{\sum \left(x-\bar{x}\right)\left(y-ȳ\right)}{\sqrt{\sum {\left(x-\bar{x}\right)}^{2}*\sum {\left(y-ȳ\right)}^{2}}} \end{array}$$
  where *r* is the correlation coefficient, *x* is the task-component (or rest-component), *y* is the template, $\bar{x}$ is the mean of the task-component (or rest-component), ȳ is the mean of the template.
- Step 3: For each channel, we calculated the signed *R*^2^ (Wonnacott and Wonnacott, 1972) index between the task-CC set compared and rest-CC set, as shown in Equation (3)
  (2) $$\begin{array}{r} G=\frac{(\sum \left(q\right)+\sum \left(t\right){)}^{2}}{2\times n} \end{array}$$
  (3) $$\begin{array}{r} R^{2}=\frac{{\left(\sum q\right)}^{2}/n+{\left(\sum t\right)}^{2}/n-G}{\sum q^{2}+\sum t^{2}-G} \end{array}$$
  where *q* is the task-CC vector, *t* is the rest-CC vector, *n* is the dimension of the task-CC (or rest-CC) vector. Then the *R*^2^ index was signed. A positive *R*^2^-value means a negative potential and a negative *R*^2^-value means a positive potential.

For example, we analyze *chan*_25 of *sub*_1 with 34 good trials. Using step 2, we can obtain 34 task-CC and 34 rest-CC. By comparing these task-CC set and rest-CC set in step 3, we can obtain a single *R*^2^-value for *chan*_25.

For each channel, a *p*-value was estimated using a balanced, one-way, analysis of variance for the task-CC set compared with rest-CC set. Each *p*-value was Bonferroni corrected to account for multiple comparisons across channels. Channel with *p* < 0.01 was ETAM significant channel.

#### 2.4.2. Electrocorticographic frequency alteration mapping, EFAM

As the wrist holding time was about 1 s, we found a sustained 1 s event-related desynchronization (ERD) in the low frequency band. Thus, we defined a 1 s task-segment and rest-segment for each trial. The task-segment was 1,000 ms following the EMG onset and the rest-segment was −3,500 to −2,500 ms. We calculated the power spectral density (PSD) of each segment using the fast Fourier transform method with a 1 s Hanning window. We normalized the power at each frequency for each segment in two steps (Equation 4): elementwise divided each spectral sample by the average across the whole ensemble (i.e., the task and rest segments), and then took the log form (Miller et al., 2009).

(4) $$\begin{array}{r} \tilde{P}\left(f,\tau_{q}\right)=ln\left(P\left(f,\tau_{q}\right)\right)-ln\left(\frac{1}{N}\overset{N}{\underset{p=1}{\sum }}P\left(f,\tau_{p}\right)\right) \end{array}$$

where *P* is the original PSD, $\tilde{P}$ is the normalized PSD, τ_*q*_ refers to both task and rest segments, the total number of events τ_*q*_ denote as *N*. We then calculated the sum of all normalized power values in two 25 Hz bands. Because of the power law form of the ECoG spectrum, without data normalization the changes at the lower end would dominate the analysis procedure. The two bands were: (1) 8–32 Hz (LFB, low frequency band) which was the mu and beta region, and (2) 66–90 Hz (HFB, high frequency band) which lied within the gamma region and avoided 50 Hz contamination. For each electrode, we calculated the activation weight by comparing the distributions of HFB or LFB values for task with the corresponding rest distributions. Each such weight (*A*) was a signed squared cross-correlation value shown in Equation (5). *A* was a measurement of the variance in power between the task and rest (Miller et al., 2007b).

(5) $$\begin{array}{r} A_{mr}=\frac{{\left(\bar{m}-\bar{r}\right)}^{3}}{|\bar{m}-\bar{r}|\sigma_{m\cup r}}\frac{N_{m}N_{r}}{N_{m\cup r}^{2}} \end{array}$$

where *A*_*mr*_ is the activation weight, $\bar{m}$ and $\bar{r}$ denote the distribution mean of task and rest segment respectively, *m*∪*r* denotes the union of the two distributions, *N* is the number of elements in each distribution, σ is the standard deviation. For each channel, a *p*-value was estimated using a balanced, one-way, analysis of variance with the normalized HFB or LFB power for the task-segment compared with rest-segment. Each *p*-value was Bonferroni corrected to account for multiple comparisons across channels. Channel with *p* < 0.01 was EFAM significant channel.

### 2.5. Brain activation maps

For topographic visualization of the mapping results, only electrodes with changes significant at the 0.01 Bonferroni-corrected level were included. Activation maps for the ETAM, HFB, and LFB in each patient were created independently. These maps were created by linear convolution with spherical Gaussian kernels (μ = 0, σ = 0.4, the diameter for each kernel was 25 mm) centered at the location of each electrode. Each of these Gaussian kernels was multiplied by the assigned weight of each electrode. The weight of ETAM was the signed *R*^2^ (Equation 3) and the weight of HFB/LFB was the value *A* (Equation 5).

### 2.6. Cortical stimulation mapping

**Figure 2:**
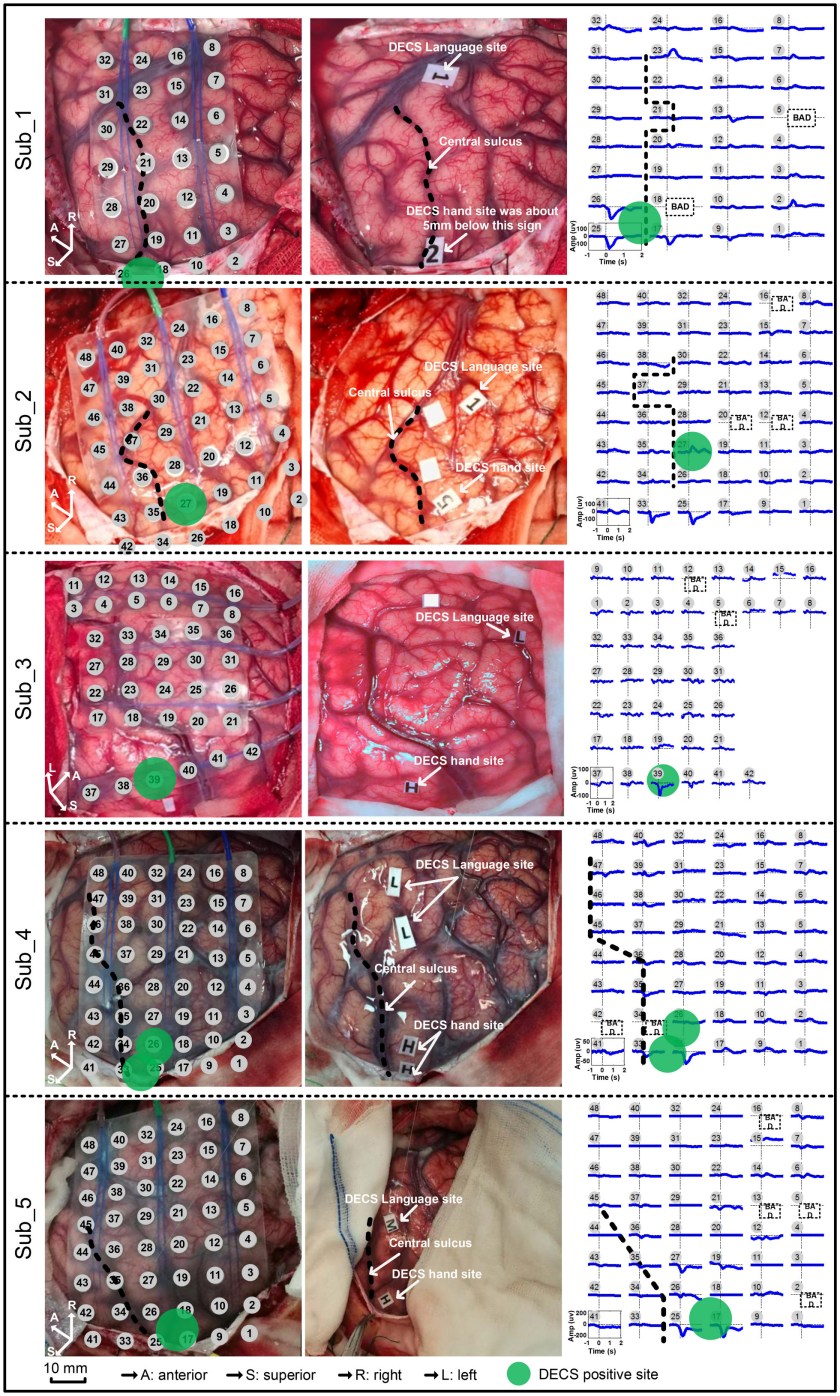
Movement-related cortical potentials (MRCPs) and its cortex distribution for all five subjects. **Left**: the electrode array on the cortex. **Middle**: the direct electrocortical stimulation (DECS) positive sites of wrist extension. **Right**: the MRCPs across all channels.

Standard DECS mapping was performed routinely as control, using a 5 mm wide bipolar electrode stimulator with 60 Hz biphasic square-wave pulse (Epoch XP, Axon Systems). Hand motor areas were identified by induced or inhibited movements of the hand after electrical stimulation. Photographs of the brain surface with motor paper label were taken. Haglund et al. (1994) illustrated that the most significant factor in preserving function is maintaining a margin of 10 mm around regions identified as DECS-positive electrodes. In this study, we drew a circle with 6mm radius centered at DECS-positive site. Electrodes involved in this circle were defined as DECS-positive electrodes (Figure 2).

### 2.7. Comparison of DECS with ETAM, LFB, and HFB maps

For each subject, we compared DECS-positive electrodes (sites with induced motor response) with electrodes that showed a significant ETAM, LFB, or HFB changes. In particular, we assessed the ETAM, LFB, and HFB electrode groups' sensitivity and specificity in identifying the DECS-positive electrodes (the true positive sites). Taken ETAM as example, the sensitivity was determined by dividing true positives (both DECS and ETAM positive electrodes) by the sum of true positives and false negatives (DECS positive but ETAM negative electrodes). The specificity was determined by dividing true negatives (both DECS and ETAM negative electrodes) by the sum of true negative and false positive (DECS negative but ETAM positive electrodes). The HFB/LFB sensitivity and specificity was calculated in the same way (Table 2).

**Table 2:** Determining sensitivity and specificity.

	**True+**	**True−**
Test+	A (true positive)	B (false positive)
Test−	C (false negtive)	D (true negative)

Sensitivity = A/(A + C); Specificity = D/(B + D)

To assess whether or not the ETAM, LFB, and HFB electrode distribution significantly overlapped with DECS-positive electrode distribution, we did the chi-square independence test with the assumption that electrode distribution were non-overlapping.

## 3. Results

Contralateral wrist motion showed well-defined MRCP in all five subjects (Figure 2). In proximity to the EMG onset, we got a distinct focalization of the motor potential (MP) nearby the central sulcus, which overlaid the hand motor representation area localized by DECS. MRCP activity peaked at ~100 ms after the EMG onset. Every subject in this study had DECS-induced hand movements. Altogether, 11 DECS-positive electrodes were found as shown in (Table 3).

**Table 3:** DECS-positive electrodes of each subject.

**Subjects**	**DECS-positive electrodes**
sub_1	chan_17 chan_25 chan_26
sub_2	chan_27
sub_3	chan_39
sub_4	chan_25 chan_26 chan_33
sub_5	chan_17 chan_18 chan_25

We analyzed the correlation among the three modalities for cortical mapping, i.e., DECS, ETAM, and EFAM. Electrodes were considered to be EFAM positive (EFAM+) if powers were significantly changed in the LFB, HFB, or both. The total number of electrodes, electrodes with functional responses, sensitivity, and specificity of each methods are summarized in Tables 4, 5. In general, no significant difference in sensitivity was found between ETAM and LFB methods, with 9 and 10 true positive electrodes, respectively. However, the ETAM was more specific compared with LFB. Explicitly, LFB for DECS-positive sites was 90.91% in sensitivity and 86.08% in specificity, while for the ETAM it was 81.82 and 95.36%, respectively. No significant high frequency alterations were found in *sub*_1, *sub*_3, and *sub*_4. There were just three channels having high frequency alterations in all five subjects. HFB for DECS-positive sites was 18.18% in sensitivity and 99.48% in specificity. Taking the LFB and HFB together, the sensitivity and specificity of EFAM positive sites for identifying DECS-positive sites was 90.91 and 86.08%, respectively.

**Table 4:** Electrodes summary.

**Electrodes summary**	**Totals**
Total no. of electrodes	205
Significant ETAM change electrodes	18
Significant HFB power change electrodes	3
Significant LFB power change electrodes	37
Either significant LFB or HFB power change (EFAM+) electrodes	37
No. of electrodes producing motor response	11
Significant ETAM and motor response electrodes	9
Significant HFB power change and motor response electrodes	2
Significant LFB power change and motor response electrodes	10
Either significant ETAM or EFAM, and motor response electrodes	10

*HFB, high frequency band; LFB, low frequency band; EFAM, electrocorticographic frequency alteration mapping; ETAM, electrocorticographic temporal alteration mapping*.

**Table 5:** Summary of statistical analysis.

**Sub**.	**Sensitivity (%)**				**Specificity (%)**			
	**ETAM**	**LFB**	**HFB**	**EFAM+**	**ETAM**	**LFB**	**HFB**	**EFAM+**
1	100	100	0	100	88.89	100	100	100
2	100	100	0	100	93.18	79.55	97.73	79.55
3	100	100	0	100	100	84.61	100	84.62
4	66.67	66.67	0	66.67	97.67	90.7	100	90.70
5	66.67	100	66.67	100	95.12	80.49	100	80.49
Total	81.82	90.91	18.18	90.91	95.36	86.08	99.48	86.08

*HFB, high frequency band; LFB, low frequency band; EFAM, electrocorticographic frequency alteration mapping; ETAM, electrocorticographic temporal alteration mapping*.

**Figure 3:**
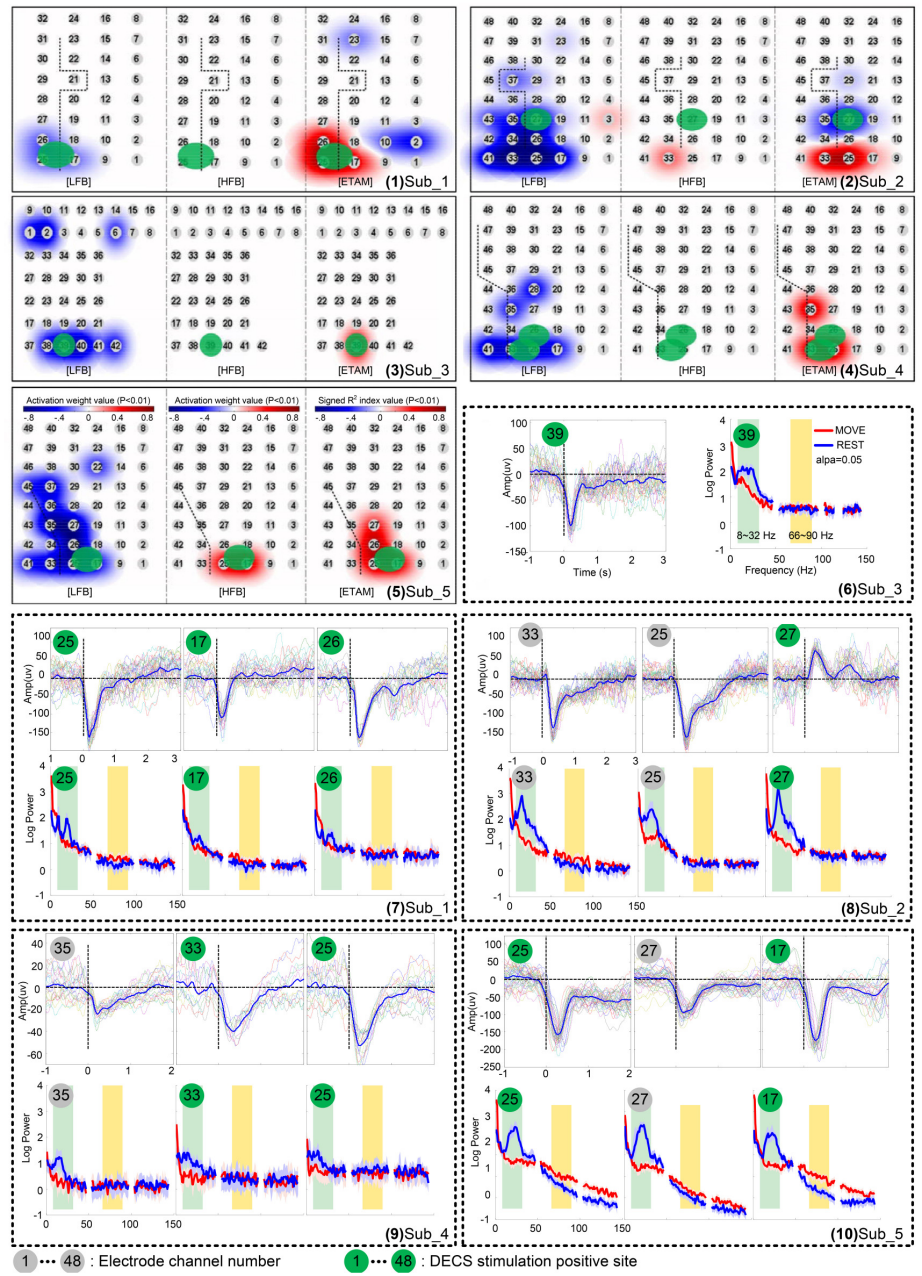
Cortical activation for wrist extension motion during awake craniotomy of five subjects. (1–5) topographic distribution maps. Electrode locations are signed in numbers with a gray cycle. For each subject, the left panel is the low frequency band (LFB) results, middle panel is the high frequency band (HFB) results, and the right panel is the ETAM results. (6–10) comparison of movement-related cortical potentials (MRCPs), low frequency band (LFB, 8–32 Hz) spectral alteration, and high frequency band (HFB, 66–90 Hz) spectral alteration. Electrode numbers with green cycle indicated DECS-positive electrodes.

Figure 3 (1–5) shows the activation maps of five subjects. A highly generalized response was found for motor tasks across individual subjects. Decreases were found in LFB while increases were found in HFB. Topographic distribution of ETAM results were more concentrated than the distribution of LFB power changes. HFB showed no somatotopical information for all subjects except *sub*_5. Further, analyzing the MRCP, LFB, and HFB for each single channel, results are shown in Figure 3 (6–10). Gamma band changes were not significant among subjects, and only *chan*_33 of *sub*_2 and *chan*_25, *chan*_17 of *sub*_5 were found with significant gamma changes. All MRCP significant channels showed significant low frequency band changes after the motion onset.

The chi-square independence test results showed that the ETAM, LFB, and HFB electrode distributions were significantly overlapped with the DECS-positive electrode distributions. Specifically, ETAM χ^2^ = 68.08, LFB χ^2^ = 36.68, HFB χ^2^ = 11.94 (α = 0.001, χ^2^ = 10.83). Cross-validation for ETAM was done for all possible combinations of the test-template and test-data (i.e., to test one subject, the test-template was the mean of the representative grand average MRCP of the other four subjects). Cross-validation results showed that ETAM for DECS-positive sites was 81.82% in sensitivity and 94.33% in specificity.

## 4. Discussion

In this work, a novel ETAM method targeting at intraoperative brain surgery was proposed and validated. Neurosurgeons could apply ETAM as an alternative technique to identify the motor cortex during awake craniotomy. Over the past decade, researches pay great attention to understand how brain electrical signals (i.e., EEG and ECoG) correlate to motor, sensory, and language functions. Generally, three territories were investigated for function localization. The first one is the utilization of spectral alteration, such as ERD/ERS (Miller et al., 2014), LFB/HFB (Miller et al., 2007b), and most recently, the broadband spectral changes (Miller et al., 2009, 2014); The second one is the utilization of temporal alteration, which is disclosed by averaging EEG/ECoG data with respect to the task onset from the background oscillatory, such as movement-related cortical potentials which are proved as a promising feature for function localization (Ikeda et al., 2002); the third one is the utilization of signal coherence, which depends on the power and phase dynamics among different frequency components from one electrode (e.g., amplitude phase coupling Fries, 2005). For the clinical application, Leuthardt et al. firstly used the term of electrocorticographic frequency alteration mapping (EFAM) for the purpose of motor cortex localization (Leuthardt et al., 2007), which belongs to the first territory. In this study, we gave the proposed MRCP-based mapping method as ETAM which belongs to the second territory. ETAM results were consistent among different subjects, with sensitivity varying from 66.67 to 100% and specificity varying from 88.89 to 100%. Our results indicated that ETAM would be a clinical and technical useful and reliable method.

This study confirmed the previously proposed notion that ECoG MRCPs can be a useful tool for eloquent cortex mapping. Motor cortex regions defined through ETAM in our study correlated well with regions that defined through DECS. MRCPs reflect discharges from large populations of neurons linking specific aspects of sensory and cognitive processing. Scalp MRCPs are susceptible to the spatially integrating properties of cerebrospinal fluid, skull and skin. Compared with scalp MRCPs, intracranial MRCPs (iMRCPs) extracted from ECoG have much larger (up to 150 microvolt) voltage and higher spatial resolution. iMRCP components are often visible in very few adjacent electrode contacts and span a few square centimeter with steep amplitude gradients, suggesting close proximity to the cortical generators (Breshears et al., 2010; Roland et al., 2010; Ogawa et al., 2014; Taplin et al., 2016). With a systematical analysis of MRCPs, researchers can understand models of these cognitive processes and locate the brain regions that implicate specific cognitive processes. We extracted large MRCP amplitudes (up to 200 uV, Figure 1) directly from the cortex surface, and had a good cross-validation sensitivity (81.82%) and specificity (94.33%) for identifying sites with positive DECS hand motor responses. These anatomic data were acquired in several minutes without the risks of seizure and after discharges caused by DECS. Especially, all experiments were performed intraoperatively which confirmed its clinical viable possibility. ETAM method proposed in this study provided a enormous amount of useful and low risk information. Regarding to the close correlation of the ETAM with DECS maps and short time requirements for information accrual, ETAM could be a powerful adjunct method for delineating the cortex in conjunction with DECS.

Many studies show that complex actions are associated with more robust brain responses than simple actions. Functional near-infrared spectroscopy (fNIRS) and functional magnetic resonance imaging (fMRI) studies indicate that complex tasks are associated with greater hemodynamic changes than simple tasks (Kuhtz-Buschbeck et al., 2003; Wei and Luo, 2010; Lisa Holper, 2011). Although, the relationship between the neural activity types and the oxygen level is complicated, cerebral local oxygenation are closely related to the neural activity (Lenkov et al., 2013; Chernov et al., 2016). By using transcranial magnetic stimulation (TMS), higher motor-evoked potential (MEP) amplitude is found with more complex task, indicating an increased corticospinal excitability with increased task-complexity (Kuhtz-Buschbeck et al., 2003; Roosink and Zijdewind, 2010). Electroencephalography (EEG) studies show that sensorimotor rhythms (SMR) elicited during complex actions can be more reliably detected comparing with simple actions (Gibson et al., 2014). ECoG studies have found no evidence for increases in gamma power in subjects discriminating the strength of a somatosensory stimulus (Menon et al., 1996). Aoki et al. (1999) indicated that different tasks generate different ECoG patterns, and negligible increases in gamma power occur with simple wrist movement. Similarly, this task-dependent changes phenomenon in gamma activity has been investigated in non-human primates (Sanes and Donoghue, 1993; Baker et al., 1997; Donoghue et al., 1998). We further confirmed this task-dependent changes phenomenon intraoperatively. With simple wrist extension movement, gamma changes were rarely found (further analyzing these data, only *chan*_33 of *sub*_2 and *chan*_25, *chan*_17 of *sub*_5 were found with significant gamma changes), and the gamma sensitivity for identify DECS-positive sites was only 18.18% for all five subjects. Thus, in specific activation, i.e., simple wrist movement, using gamma changes for motor cortex location was found to be less valid.

The relation between low frequency ([8–30] Hz) ERD and MRCPs is controversial. ECoG recordings from primary sensorimotor (M1-S1) show that MRCP and ERD responses originate in similar cortical regions and share some common timing features, but the magnitude and spatial distribution of the two responses appear to be independent (Toro et al., 1994). In an EEG study, alpha ERD reflects changes in wide cortical sensorimotor areas, whereas MRCPs represent mainly task-specific responses of the supplementary motor area (SMA) and contralateral M1-S1 (Bittar et al., 1999). Results of this study indicated that LFB and MRCPs responded in similar cortical regions, but LFB seemed to have a broader distribution than MRCPs. Sensitivity of the MRCPs and LFB for identifying DECS-positive sites had no obvious difference, but MRCPs tended to be more specific (94.33%) than LFB (86.08%).

The intraoperative condition is stricter than the extraoperative condition. Patients are always anxious and frail, and the time is limited for safety reasons. More complexity in tasks means more conscious and active involvement from patients. But more conscious means more time for awaking from anesthesia. Generally, complex tasks can only be performed with well-cognitive ones. Elderly person, children, and patients with cognition disorders would be extremely difficult to perform the required complex tasks. To reduce the cognitive complexity, patients were instructed to execute single wrist extension cued by the surgeon many times without other tasks between trials, and all patients could easily and successfully perform this intraoperative wrist motion task.

In order to further verify clinical applicability of ETAM, cross-validation was done for all possible combinations of the test-template and test-data, and the results was 81.82% in sensitivity and 94.33% in specificity. Comparing with the results using the template which was defined by averaging the representative MRCP chosen from all five subjects (Figure 1), the cross-validation results were the same in sensitivity (81.82 vs. 81.82%) while slightly decreased in specificity (94.33 vs. 95.36%). Thus, we only needed to calculate the template once, which was clinically practicable and was a promising way to shorten the operation time. The sensitivity for identifying DECS-positive sites may be improved when the template is calculated from the same subject, but it will need additional trials and may not be the optimal choice when taking limited operation time into consideration. The movement onset was visually defined in this study in order to exclude the noisy trials, but it could be automatically defined by estimating the onset when the rectified EMG signal amplitude crossed a certain threshold (e.g., set threshold as the one tenth of maximum EMG amplitude) (Niazi et al., 2011). However, using the EMG amplitude for template onset definition was an indirect method. There may have significant EMG changes without significant MRCP because of additional cognitive process or unknown noise. For rapid and reliable function mapping, a movement detector based on MRCP is needed for real-time detection of movement initiation. Prior studies of our groups showed that the single trial MRCP induced by upper and lower limbs could be detected in real-time, which were based on EEG study and aimed for a closed-loop brain-computer interface application. In these studies LPP-LDA (Jochumsen et al., 2015) and classic matched filter (Jiang et al., 2015) were applied as MRCP detector, and we could use similar strategies in a further work toward a real time function mapping based on ETAM method.

To obtain a friendly interactive interface and present the ETAM results, we applied a weighted spherical kernel to each electrode, and projected the summed results on a grid map with electrode number [Figure 3 (1–5)]. This procedure allowed surgeons to intuitively appreciate the eloquent cortex associated with the relevant function.

Recording reliable ECoG signal is not easy and is one of the main limitation hindering its clinical application, especially in the operation room where lot of surgical instrument produce unknown noise. For example, the spectral power and MRCP amplitude of *sub*_4 was much smaller than other subjects [Figure 3 (9)]. Further analyzing the raw data, we found an extraordinary large 50 Hz power-line interference which even led to the amplifier saturation some times. However, some clear MRCPs trials could still be found in sub_4, and the contaminated trials were visually excluded. By analyzing 19 uncontaminated trials, the ETAM for DECS-positive sites was 66.67% in sensitivity and 97.67% in specificity, while for the LFB it was 66.67 and 90.7% respectively. ETAM had better specificity than LFB, indicating that ETAM may be more robust in noisy environment.

**Figure 4:**
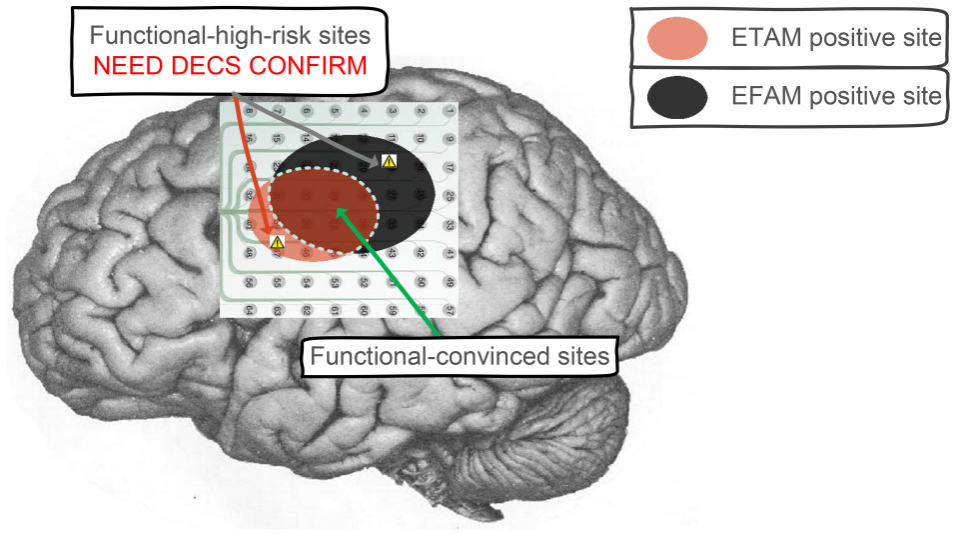
Clinical application to combine ETAM and EFAM together. Both ETAM and EFAM positive electrodes were regarded as functional-convinced sites, electrodes with opposite results were regarded as functional-high-risk sites. The functional-convinced sites need to be protected, while additional DECS procedure need to be performed on the functional-high-risk sites for function confirmation.

Dalal et al. holds the view that it is not wise to restrict our understanding of brain dynamics exclusively to higher frequency content, and the brain dynamics need to be examined across several frequency bands (Dalal et al., 2011). In specific condition of this study, gamma band alteration was negligible. The temporal and spectral alteration indicates different brain physiological mechanisms, which may represent different aspects of motor cortex activation. In this study, ETAM method contained [0.05–3] Hz information while EFAM method contained LFB ([8–32] Hz) and HFB ([66–90] Hz) information. For motor cortex mapping, the ETAM and EFAM methods shared some common positive electrode results while still had some opposite results, e.g., *chan*_2 of *sub*_1 was ETAM-positive but EFAM-negative. These opposite results may provide additional information to the neurosurgeons. Clinically, both ETAM and EFAM positive electrodes might be regarded as functional-convinced sites, and electrodes with opposite results might be regarded as functional-high-risk sites, as shown in Figure 4. The neurosurgeons need to protect the functional-convinced sites while be careful about the functional-high-risk sites, and additional DECS procedure might be performed on these functional-high-risk sites for function confirmation. As mentioned by Towle et al. that a formidable challenge will be to discriminate between necessary and ancillary areas of cortical function, which are probably not discrete entities, but rather points along a continuum (Towle et al., 2016). When combining the results of ETAM and EFAM together, the sensitivity and specificity for identifying DECS-positive sites were 90.90 and 84.53%, respectively. The combining sensitivity was the same comparing with EFAM (90.90%), but the specificity was lower than EFAM (86.08%) and ETAM (94.33%). Thus, combining the two methods might not improve the sensitivity and specificity, but might be a promising way to separate the functional area into necessary and ancillary areas. Technically speaking, when setting the signal sampling amplifier with a DC analog cut off, researchers can obtain both fine MRCP and LFB/HFB signal.

## 5. Conclusion

In this work, a novel ETAM method based on MRCP was proposed for intraoperative mapping. The feasibility and resolution of the proposed ETAM mapping was compared with clinically available DECS approach which requires active cortical stimulation. Due to the passive mapping feature of the ETAM methodology, the proposed method would significantly reduce the risk of inducing seizures during surgery. Moreover, by comparison with the state-of-the-art EFAM method, ETAM demonstrated a better performance in specificity while showed a comparable performance in sensitivity in condition of simple task. For clinical application, as the temporal and spectral alteration indicates different brain physiological mechanisms, combination of the ETAM with EFAM would provide neurosurgeons with more reliable information for a better operation planning.

## Ethics statement

This study was carried out in accordance with the recommendations of Ethics Committee of Huashan Hospital, Fudan University with written informed consent from all subjects. All subjects gave written informed consent in accordance with the Declaration of Helsinki. The protocol was approved by the Ethics Committee of Huashan Hospital, Fudan University.

## Author contributions

LC and YM conceived the study. LY, DZ, XS, and LC designed the experiment. ZW, TX, and LC conducted the experiments. ZW and TX analyzed the data. ZW, TX, and LY co-drafted the paper. DF, YM, and XZ coordinated the work.

### Conflict of interest statement

The authors declare that the research was conducted in the absence of any commercial or financial relationships that could be construed as a potential conflict of interest.
